# Physical Activity and Motor Competence in Preschool Children

**DOI:** 10.3390/children8040305

**Published:** 2021-04-16

**Authors:** Vladimir E. Martínez-Bello, Isaac Estevan

**Affiliations:** 1 COS Research Group, Body, Movement, Music and Curricular Practices, University of Valencia, 46022 Valencia, Spain; 2 Department of Teaching of Musical, Visual and Corporal Expression, University of Valencia, 46022 Valencia, Spain; isaac.estevan@uv.es; 3 Activitat Física i Promoció de la Salut (AFIPS) Research Group, University of Valencia, 46022 Valencia, Spain

## 1. Introduction

Longitudinal evidence has demonstrated that engagement in physical activity (PA) and the development of motor competence (MC) have numerous tangible health and developmental benefits [1]. Preschool children are thus in an important stage for physical, mental and social development [2]. Likewise, early childhood (the period encompassing the preschool ages, 3–5 years old) is considered to be an important phase for developing fundamental movement skills (FMS) [3], apparently by attaining adequate levels of PA [1]. The research evidence suggests that most preschool children do not accumulate adequate levels of PA [4], which directly impacts MC, with low levels at the primary school age [5,6,7]. The negative effects of poor PA associated with low MC seem to be affected by individual characteristics and the physical and social environment [5,8,9]. This is particularly important for low SES children living in low-income areas, girls, individuals with no siblings at home and/or the relative age effect (RAE), since these correlates seem to influence children’s PA and MC [5,10,11,12].

This Special Issue is devoted to systematic evaluations of PA and MC, all of which have an impact on preschool children’s knowledge of themselves and their movement possibilities. We here summarize the most important findings of the different contributions published in this Special Issue.

## 2. Contributors

Taking into account that early childhood education (ECE) environments have great potential to promote PA and reduce SB in young children, Lahuerta-Contell et al. [13] examined the PA levels of Spanish preschoolers (*n* = 116; 4.3 years old; 48.7% girls) during school hours and the role of structured movement sessions and recess time in the MVPA levels during school hours, as well as the adherence to specific MVPA recommendations. The authors found that preschoolers engaged in very little total PA and MVPA (girls were significantly less active than boys), falling far short of compliance with the international and national recommendations [13]. The authors also found that children showed significantly higher MVPA levels on days with structured movement sessions and that the contribution of these sessions to total MVPA was significantly higher than that of recess time. They thus advocated that ECE should complement structured movement opportunities with models that increase opportunities for PA throughout the school day.

Within the framework of the role of ECE environments, Fathirezaie et al. [14] used a quasi-experimental design to study the effect of different environmental contexts, such as indoor (kindergartens) and outdoor (nature schools) spaces, on the MC and social maturity of Romanian preschool children (*n* = 30; 5.5–6.5 years old; 56.6% girls). The authors found that a developmentally-rich context such as that of nature schools can provide assured, confident and enriching opportunities for the development of motor proficiency and social maturation in almost all preschool children [14]. Taking into account that the person, the environment and the task are tightly integrated during childhood, pedagogical ideals suggest that many outdoor environments should be modified to respond to the needs of the children and to enrich their daily outdoor activity.

Even though school settings can be characterized by particular aspects, such as urban or nature schools, educational systems must be globally organized by standard aspects that integrate children appropriately in groups such as chronological age [11]. However, such an environmental context might make it possible for some preschool children in the same group to be six–eleven months younger than their counterparts, with consequences on their physical, mental and/or socio-emotional development. Aiming to examine 4 year-old Spanish children’s MC according to the RAE, Navarro-Patón et al. [11] studied 132 children classified into four groups in terms of the trimester they were born (January–March, Q1; April–June, Q2; July–September, Q3; October–December, Q4). Applying the Movement Assessment Battery for Children-2 (MABC-2), they found boys outperformed girls in overall MC, and those in Q1 exhibited higher manual dexterity, balance and overall MC than the rest of the children. This study found that Q1 children are at an advantage in MC over those in Q3 and Q4, which might have educational implications in terms of curricular progression, motivation, free play, time of practice and/or evaluation.

Preschool is not mandatory in most countries, so the educational setting can also be seen as an expanded social environment in which children live independently and autonomously for the first time. This onset of interpersonal relationships is linked to mental and physical health and interdisciplinary competence, because preschoolers learn to develop their own social strategies and new rules, behave cooperatively and prosocially towards peers and find resolutions to conflicts [2]. In ECE, it seems boys usually engage in individual forms of play including competitiveness, while girls experience cooperative forms of play [2]. For the purpose of analyzing the relationship between friendship (proxy-reported by teachers and parents) and preschool children’s MC in boys and girls by age, Herrmann et al. [2] studied 548 Swiss children (4–6 years old; 49.1% girls) who performed the Children Basic Motor Competencies test (MOBAK-KG). As expected, the social integration (reported by teachers) and social relationship skills (reported by parents) were higher in older children than in their younger counterparts, who also exhibited lower MC than the older children. Social relationships were associated with MC, especially in boys. In other words, when considering sex, social integration and relationships were linked to boys’ MC but not to girls’. With these results in mind, the authors suggested that the preschool setting could be seen as a context in which social peers’ relationships are fostered, which in turn enhances children’s MC but requires close attention in the case of girls. This is important, because the positive or upward spiral of the association between MC, PA, mental health and social integration should be cultivated not only in a subject-specific way but also in an interdisciplinary way, especially in girls, who seem to be at a higher risk of consequences to their health.

Due to the relationship between PA and MC, the appropriate levels of MC are not only achieved through natural development and maturation, but through continuous interaction with a stimulating and supportive social and physical environment [15,16]. In addition to the educational setting in which children interact with friends and classmates, siblings are another group of significant others for children that can influence preschoolers’ motor development [10]. Honrubia-Montesinos et al. [17] studied Spanish preschoolers’ (*n* = 300; 3–5 years old; 44% girls) motor development by sex, age, number of siblings, prematurity and participation in extracurricular PA. The authors found that in boys and girls at 3 and 4 years of age, boys scored significantly higher than girls in locomotor and object control, but at 5 years of age, these differences in favor of boys were only maintained in object control, and not in locomotor skills. Even though primary school boys tend to outperform girls in object control skills [5], preschoolers exhibited similar MC, regardless of sex at age 3 and 4, which is not in agreement with the findings of Herrmann et al. [2], who found that boys showed higher MC in movements involving balls (object movement), and girls achieved higher MC in whole-body movements (self-movement). The authors concluded that extracurricular activities in which children practice PA could have a positive impact on motor development. However, prematurity and being an only child were not decisive in motor development [17]. According to these findings, further research is needed to understand the influence of siblings in preschoolers’ MC by sex.

In this line of study, in a Portuguese context, Rodrigues et al. [12] studied associations between the influence of siblings on MC [12]. The authors studied 161 preschool Portuguese children between 3 and 6 years old (34 being only children and 125 with siblings). When preschoolers were classified as high, average or low according to their percentile score, similar MC was found for only children and siblings, regardless of the MC category level. Interestingly, more children with siblings were classified as high-MC than only children. In the European Union, 47.7% of households with offspring are characterized by having only one child, which may limit the children’s motor development [12]. When a child lives together with siblings, he/she can acquire a synergic and reciprocal role in favor of motor development, i.e., the first-born child usually engages in activities as the leader, teacher or helper of the second-/third-born child who would imitate, follow or be the learner. As evidence of the effect of having siblings appears to be exhibited around 2 years old, the effect could be higher in primary school years [18]. As a result, only children, especially girls, could be at risk of showing poor MC [10]. 

Taken together, most of these studies emphasize the idea of the leading role generally exercised by schools in promoting PA, and particularly the role of teachers and practitioners in the importance of promoting structured movement opportunities to accompany the positive development of FMS and wellbeing of girls and boys during early childhood. As shown by Alonso-Martínez et al. [19], the COVID-19 lockdown has negatively impacted the movement behavior of preschoolers, so that ECE institutions should assume greater responsibility for offering children adequate movement experiences during the pandemic.

## 3. Recommendations for Future Research

As a key period during childhood, preschool is an appropriate time for intervention as preschoolers are keen participators in PA, and new positive behaviors may be learnt to prevent negative health trajectories [20]. In addition to the proposed association between PA and actual MC, it is suggested that perceived MC is a mediator of the relationship between actual MC and PA in early childhood [21]. However, most of the studies conducted on early childhood are cross-sectional and involve limited sample sizes [20]. It is time to face prospective cohort studies analyzing the longitudinal association between actual MC and PA, including intra-individual (e.g., sex, REA, SES, perceived MC, etc.), environmental (e.g., nature, built, etc.) and social correlates (e.g., social support, existence of siblings, etc.) across preschool.

In addition, individual movement behaviors and MC can be explained according to bio-ecological theory [22] and motor development models [21]. When research questions are framed in these models, the findings can be described in a thorough and appropriate rationale, moving the topic towards a holistic perspective on the role of correlates of PA and MC for physical, mental and socio-emotional health. Study backgrounds should thus be framed into specific theories and models which might lead authors to explain the findings clearly and thoroughly, making the scientific evidence easy to understand by teachers and/or practitioners.

## 4. Conclusions

This Special Issue confirms that the correlates that impact PA and MC are not only present in primary education but are already manifested in early childhood. With this Special Issue we think that the main aim has been achieved: to increase scientific knowledge on the study of positive practices in and outside of the physical education setting in the systematic evaluation of PA and MC in the ECE context; as well as the identification of policies and programs and the role of families, teachers and practitioners, all of whom have an impact on preschool children’s knowledge of themselves and their movement possibilities. However, this pandemic could challenge our assumptions about these correlates that positively and/or negatively impact the PA and MC of young children, and schools and ECE teachers and practitioners should take a stand to promote and offer quality curricular practices.
